# Carbide-free one-zone sulfurization method grows thin MoS_2_ layers on polycrystalline CVD diamond

**DOI:** 10.1038/s41598-018-38472-9

**Published:** 2019-02-14

**Authors:** Michaela Sojková, Peter Siffalovic, Oleg Babchenko, Gabriel Vanko, Edmund Dobročka, Jakub Hagara, Nada Mrkyvkova, Eva Majková, Tibor Ižák, Alexander Kromka, Martin Hulman

**Affiliations:** 1 Institute of Electrical Engineering, SAS, Dúbravská cesta 9, 841 04 Bratislava, Slovakia; 2 Institute of Physics, SAS, Dúbravská cesta 9, 845 11 Bratislava, Slovakia; 3 Institute of Physics AS CR, Cukrovarnícka 10, 162 00 Praha 6, Czech Republic

## Abstract

The last few decades faced on the fabrication of advanced engineering materials involving also different composites. Here, we report on the fabrication of few-layer molybdenum disulfide on top of thin polycrystalline diamond substrates with a high specific surface area. In the method, pre-deposited molybdenum coatings were sulfurized in a one-zone furnace at ambient pressure. As-prepared MoS_2_ layers were characterized by several techniques including grazing-incidence wide-angle X-ray scattering, atomic force microscopy, scanning electron microscopy, Raman spectroscopy and X-ray photoelectron spectroscopy. We found out that the initial thickness of Mo films determined the final c-axis crystallographic orientation of MoS_2_ layer as previously observed on other substrates. Even though it is well-known that Mo diffuses into diamond at elevated temperatures, the competing sulfurization applied effectively suppressed the diffusion and a chemical reaction between molybdenum and diamond. In particular, a Mo_2_C layer does not form at the interface between the Mo film and diamond substrate. The combination of diamond high specific surface area along with a controllable layer orientation might be attractive for applications, such as water splitting or water disinfection.

## Introduction

Combination of different materials with complementary properties expands their application range and opens new possibilities for research. In this terms, the diamond and molybdenum disulfide thin films represent an attractive pair with a promising potential for sustainable technologies such as water disinfection^1,2^, water splitting^3^ or solar cells^4–6^.

Transition metal dichalcogenides (TMDCs) are layered materials with a formula MX_2_, where M and X correspond to a transition metal and chalcogen, respectively. MoS_2_ is recently one of the most studied TMDCs composed of covalently bonded S-Mo-S sheets which are bound by weak van der Waals forces. MoS_2_ is a semiconductor with an indirect bandgap of about 1.3 eV^7,8^, however, the monolayer MoS_2_ is a direct gap semiconductor with a bandgap of 1.8 eV^9^. This is, in particular, important for photovoltaic^7^ and photocatalytic^8^ applications due to its strong absorption in the solar spectral region.

Other applications of MoS_2_ include transparent and flexible field-effect transistors (FETs)^10,11^, photodetectors^12^, photovoltaic cells^13,14^, light-emitting diodes^15^ and catalysts^16,17^, drug delivery and biosensors^18^.

Diamond thin films have a great number of unique properties^19–21^. Indeed, its great mechanical hardness, wear resistance, chemical inertness, high thermal conductivity, optical transparency, and biocompatibility combined in one platform are advantageous for numerous application^19–21^. The diamond films are commonly used as protective coatings for cutting tools and sealing^22,23^, infrared windows and optical elements^24,25^, MEMS and electronic devices^26,27^, heater elements or heat sink^28,29^ and also in various bio-related researches^21,30–32^. In the last decade, the antibacterial effect of diamond thin films and/or diamond nanoparticles was also investigated^33,34^.

For MoS_2_ thin films, diamond may act as a mechanically stable and chemically inert carrier substrate with a high thermal conductivity which additionally can be made electrically conductive via boron doping^19–21^. Moreover, the surface of the diamond film can be structured to increase the surface area^35,36^. However, only composites of MoS_2_ with diamond-like carbon films have been reported to date^37,38^. Relatively thick films (600 nm) were fabricated by a biased target ion beam deposition technique simultaneously sputtering a MoS_2_ target and depositing DLC from CH_4_ gas^37,38^.

Here, we present fabrication of MoS_2_ thin films on diamond substrates. We are not aware of any other publication where a diamond has been used as a substrate for growing a thin MoS_2_ layer. We prepared MoS_2_ by sulfurization of thin pre-deposited Mo films with a different thickness^39^. In this method, a Mo film is annealed in vapors of sulfur at high temperatures converting Mo to MoS_2_. In the standard procedure^40–42^, at least two temperature regimes are employed during the synthesis. Sulfur evaporates from a powder source usually held at a temperature above the sulfur melting point. On the other hand, the Mo film must be heated to much higher temperatures to trigger the sulfurization. In our experiments, a simplified sulfurization process was used. The substrate and the sulfur powder were placed at the same position in the center of a one-zone furnace^39^. This enables affordable vacuum-free experimental set-up. It is important to note that the fabrication process has no impact on the diamond substrates. Molybdenum carbide (Mo_2_C) grows at temperatures above 550 °C from thin Mo films deposited on diamond^43,44^. Within the sulfurization method employed, Mo diffusion into the diamond substrate and a subsequent reaction leading to formation of molybdenum carbide was suppressed. Similarly to other substrates, it was found that the thickness of the initial Mo film determined whether the final MoS_2_ layer was aligned parallel or perpendicular to the substrate^45–47^. For the thickest MoS_2_ layers, well crystallized and densely packed MoS_2_ grains growing upright to the surface of large diamond crystallites were observed. This may have an important implication for applications deserving a large surface area.

## Experimental Section

### CVD synthesis of diamond thin films

Planar microcrystalline CVD diamond thin films were grown on the silicon substrate by plasma enhanced chemical vapor deposition (PE CVD) in the focused microwave plasma cavity resonator AIXTRON P6 with a deposition area up to 5 cm in diameter. The microcrystalline CVD diamond films were grown from H_2_/CH_4_/CO_2_ gas mixture with a flow rate of 300/15/4.5 sccm, respectively. The gas pressure was set on 60 mbar, and microwave power to 3000 W. The average temperature during 5 hour long deposition was 730 °C. The thickness of the as-prepared diamond films was 1.5 µm.

### MoS_2_ fabrication

**Figure 1:**
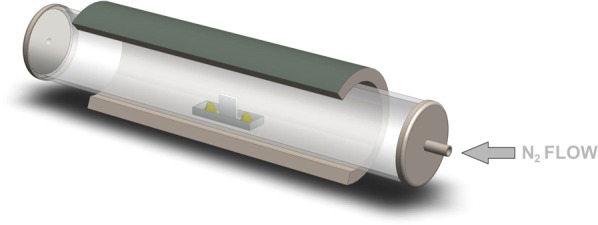
Schematic representation of the one-zone sulfurization method. A Mo coated substrate in a quartz boat is placed in the middle of the furnace along with the sulfur powder. A typical sulfur load was 3 g.

Firstly, molybdenum films were prepared by DC magnetron sputtering in Ar atmosphere (10^−3^ mbar) from a molybdenum target at room temperature. The DC power and emission current were 140 W and 0.3 A, respectively. The thickness of as-prepared Mo films was controlled by the rotation speed of a sample holder. Further, MoS_2_ films were prepared by sulfurization of pre-deposited Mo layers in a custom-designed CVD chamber. In particular, a Mo layer was annealed in sulfur vapors in a nitrogen atmosphere at ambient pressure. A one-zone furnace has the substrate and sulfur powder placed at the same position and temperature in the center of furnace as shown in Fig. 1.

The furnace was connected to a nitrogen supply and heated at a rate of 25 °C.min^−1^ to the process temperature. The sulfurization temperature and time were 800 °C and 30 min, respectively. After this process step, the temperature was ramped down to 200 °C at a rate of 20 °C.min^−1^, followed by a spontaneous cooling.

### Chemical composition analyses

Raman measurements were provided by confocal Raman microscope Alpha 300R (WiTec, Germany) using a 532 nm excitation laser line. The laser power was kept below 1 mW to avoid any beam damage. The scattered light was collected by 50× (NA = 0.8) microscope objective and detected by a cooled CCD camera. For dispersing the scattered light, a grating with 1800 gr/mm was used. The spectral resolution of the entire Raman system is about 0.75 cm^−1^. All the spectra were taken at ambient conditions.

XPS (X-ray photoelectron spectroscopy) spectra were recorded using Thermo Scientific K-Alpha XPS system (Thermo Fisher Scientific, UK) equipped with a micro-focused, monochromatic AlKα X-ray source (1486.6 eV). X-ray beam of 400 µm in diameter was used at 6 mA × 12 kV. The spectra were acquired in the constant analyzer energy mode with the pass energy of 200 eV for the survey. Narrow regions were collected using the snapshot acquisition mode (150 eV pass energy), enabling rapid collection of data (5 s per region). Charge compensation was achieved with the system flood gun that provides low energy electrons and low energy argon ions (20 eV) from a single source. Thermo Scientific Avantage software was used for digital acquisition and data processing. Spectral calibration was performed using an automated calibration routine and the internal Au, Ag and Cu standards.

### Morphological and structural analyses

Scanning electron microscopy (SEM) was performed using FEI FEG250 with resolution of 1.2 nm equipped with SE and BSE detector. The surface morphology of the diamond and MoS_2_ films was probed by atomic force microscopy (AFM) in a tapping mode (Bruker, Dimension Edge), using an etched silicon probe (Bruker, RTESPA - 300).

The crystallographic structure and orientation of the films were examined by X-ray diffraction (XRD) (CuKα) in the classical Bragg–Brentano and in the grazing-incidence configuration using BRUKER AXS D8 DISCOVER diffractometer with a rotating Cu anode.

The grazing-incidence wide-angle X-ray scattering (GIWAXS) measurements were performed using Nanostar system (Bruker AXS, Germany) equipped with IμS microfocus Cu X-ray source (λ = 0.154 nm). The parallel X-ray beam after Montel optics was further collimated using evacuated pinhole collimator equipped with two 550 µm pinholes separated by 1 m. The grazing-angle of incidence of X-ray beam on the sample was set to 0.8°. Reciprocal space maps were measured using an image plate detector at a sample-to-detector distance of 80 mm. All GIWAXS measurements were performed in fully evacuated chamber.

## Results and Discussion

**Figure 2:**
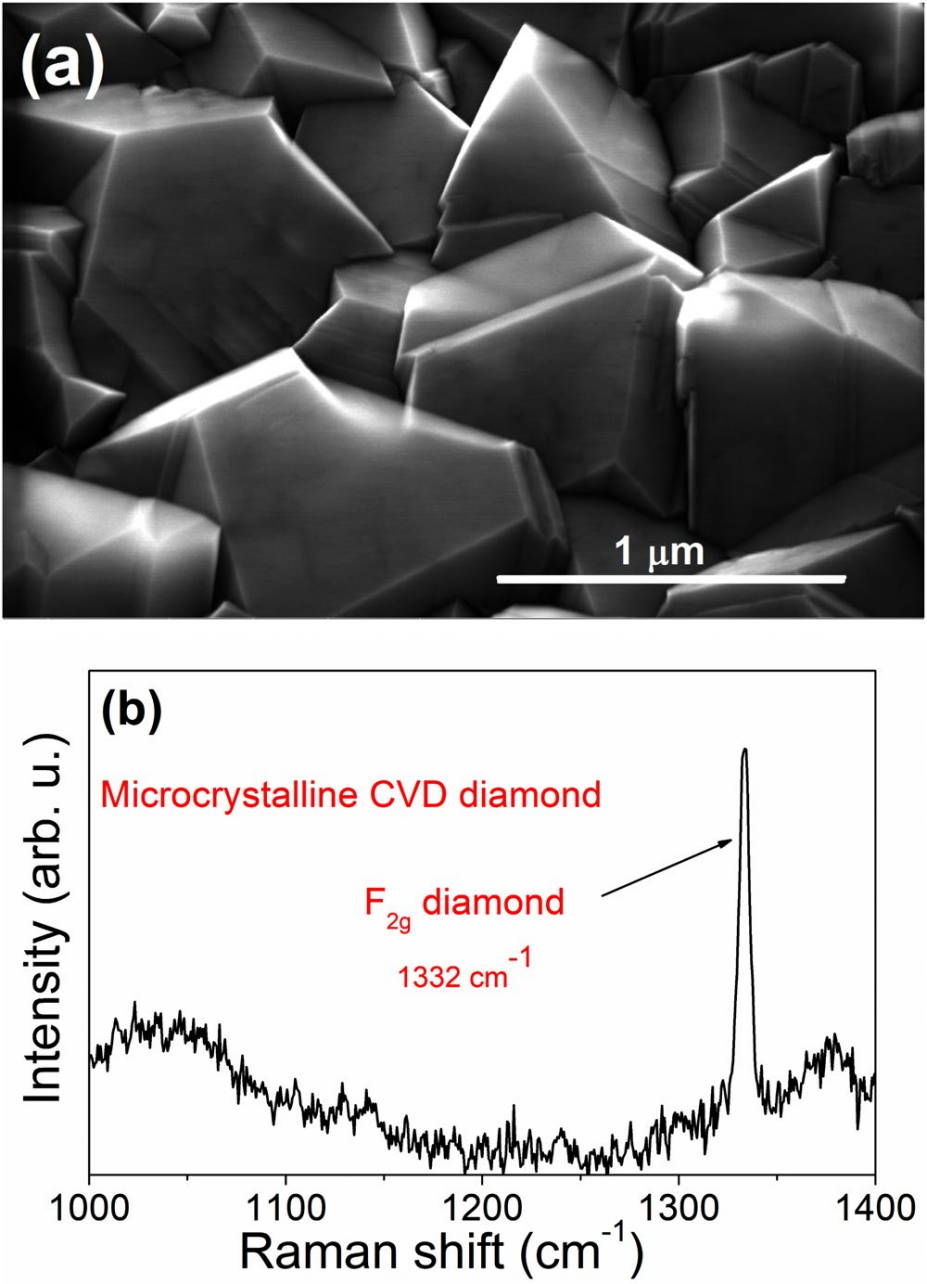
(**a**) SEM image and (**b**) Raman spectrum of a microcrystalline CVD diamond film.

SEM micrograph of a microcrystalline CVD diamond thin film used as a substrate for the deposition of Mo films is shown in Fig. 2a. The film consists of well faceted diamond grains with an average size of approximately 1 µm. The crystallographic orientation of the grains is random. The Raman spectrum (Fig. 2b) displays the dominant Raman active mode at 1332 cm^−1^ (F_2g_) corresponding to a vibration of the sp^3^ diamond lattice^48^.

**Figure 3:**
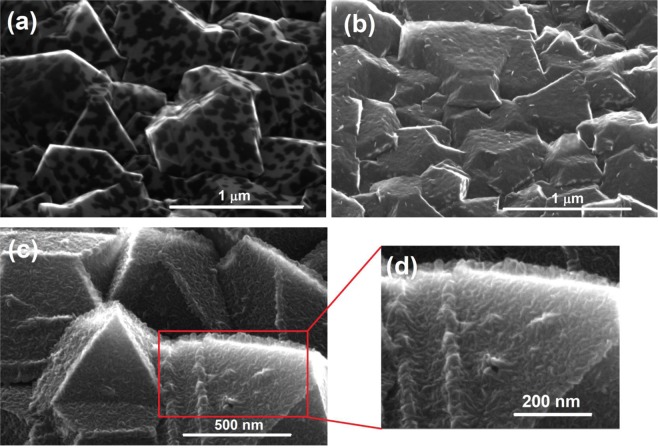
SEM images of MoS_2_ layers grown from (**a**) 1 nm, (**b**) 3 nm and (**c**,**d**) 6 nm thick Mo films deposited on the microcrystalline CVD diamond substrate. In (**d**), standing MoS_2_ flakes are seen on the edge of a diamond crystallite.

SEM images of the diamond substrate after the growth of MoS_2_ are shown in Fig. 3. It was found that the MoS_2_ morphology is determined by the initial thickness of the seeding Mo film. The MoS_2_ layers, sulfurized from the thinnest Mo film (1 nm thick), consist from isolated or partially interconnected islands (flakes) having hexagonal shape in some cases (Fig. 3a). In the case of the 3 nm thick Mo seeding film, the as-grown layer seems to be continuous covering all the facets of the diamond substrate (Fig. 3b). Finally, the sulfurization of the thickest Mo films (6 nm) reveals a significant change in the MoS_2_ preferential growth mode (Fig. 3c,d). We observe thin flakes very likely standing upright on the diamond facets (Fig. 3d). With the increasing Mo thickness, we observe the transition from separated islands through continuous films to vertically aligned flakes. The initial Mo thickness plays an important role in the c-axis orientation exactly as in the case of the MoS_2_ films grown on the flat substrates^45,47,49,50^. While MoS_2_ grown from the thinner Mo has c-axis perpendicular to the substrate plane, the thicker Mo leads to c-axis rotated by 90°.

**Figure 4:**
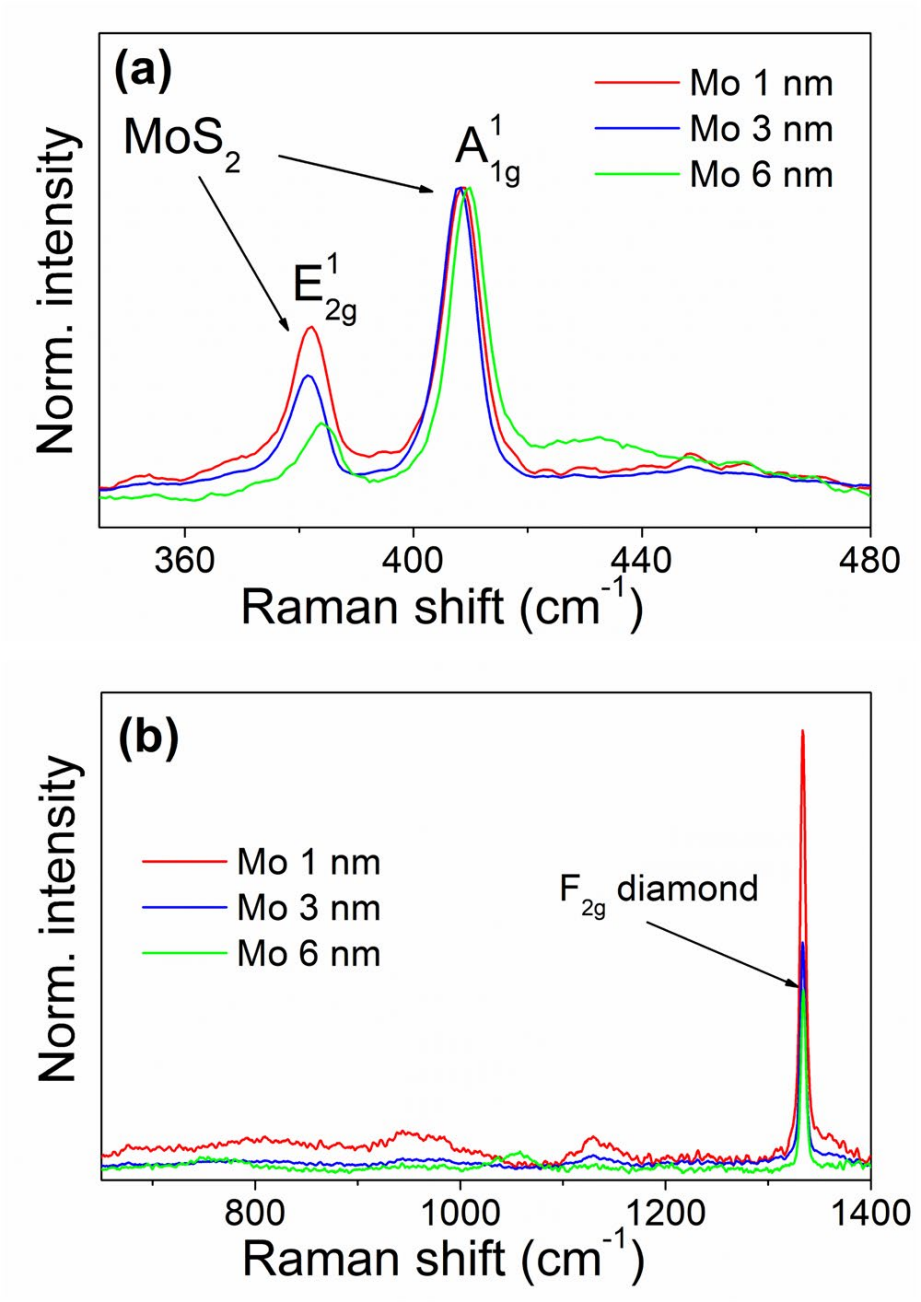
Raman spectra of MoS_2_ layers grown from 1, 3 and 6 nm thick Mo film on a microcrystalline CVD diamond substrate with (**a**) the two dominant MoS_2_ peaks and (**b**) the diamond line.

Raman spectra shown in Fig. 4 confirmed the conversion of Mo to MoS_2_. Figure 4a displays a part of the spectrum with lines belonging to 2H-MoS_2_, namely the E_2g_ mode at around 382 cm^−1^ and the A_1g_ mode at ~407 cm^−1^^51^. The spectra are normalized to the intensity of the A_1g_ mode. We observe a gradual decrease of the mode’s intensity with an increasing the initial thickness of the Mo film. In agreement with the SEM images, this dependence points out to a different crystallographic unit cell alignment of the individual MoS_2_ layers with respect to the substrate. In contrast to thinner ones, the individual MoS_2_ layers may grow perpendicularly to the substrate for the thickest MoS_2_ layer^45^.

The position of the E^1^_2g_ line for the sample grown from the thickest Mo film is blue shifted by about 3 cm^−1^ compared to other samples. Also the line becomes asymmetric in shape. This is likely a consequence of the strain which both shifts the line and lifts the degeneracy of the E^1^_2g_ mode making it asymmetric^52,53^.

For the MoS_2_ samples having 1–6 layers, the distance between the E^1^_2g_ and A^1^_1g_ lines is a measure of the layer thickness^54^. However, our samples are too thick for this method to be applicable. The distance between the two lines is more than 25 cm^−1^ for all our samples. Even though we did not measure the actual thickness of the layers, it can be estimated from published data relating the thickness of the initial Mo film and final MoS_2_ layer. It was found that the latter is thicker by a factor of 3.5–4 than the former for horizontally aligned MoS_2_ layers^45,55^. This means that the MoS_2_ layers grown from 3 nm thick Mo films are about 10 nm thick. For vertically aligned layers, the thickness is a matter of definition. At least, it can be estimated from Fig. 3d that the MoS_2_ flakes are about 20 nm tall. As expected from the thickness consideration, we did not observe the photoluminescence in our samples. That is strong for monolayer MoS_2_ and its intensity decreases rapidly with the increasing layer thickness^56,57^.

As shown in Fig. 4b, besides the change in intensity, the Raman spectrum of the diamond substrate does not change with the thickness of the MoS_2_ layer. More important is the fact that no lines from the molybdenum carbide (Mo_2_C), were observed in the range between 800 and 1000 cm^−1^, where the Mo_2_C Raman lines are the most intense^58,59^.

**Figure 5:**
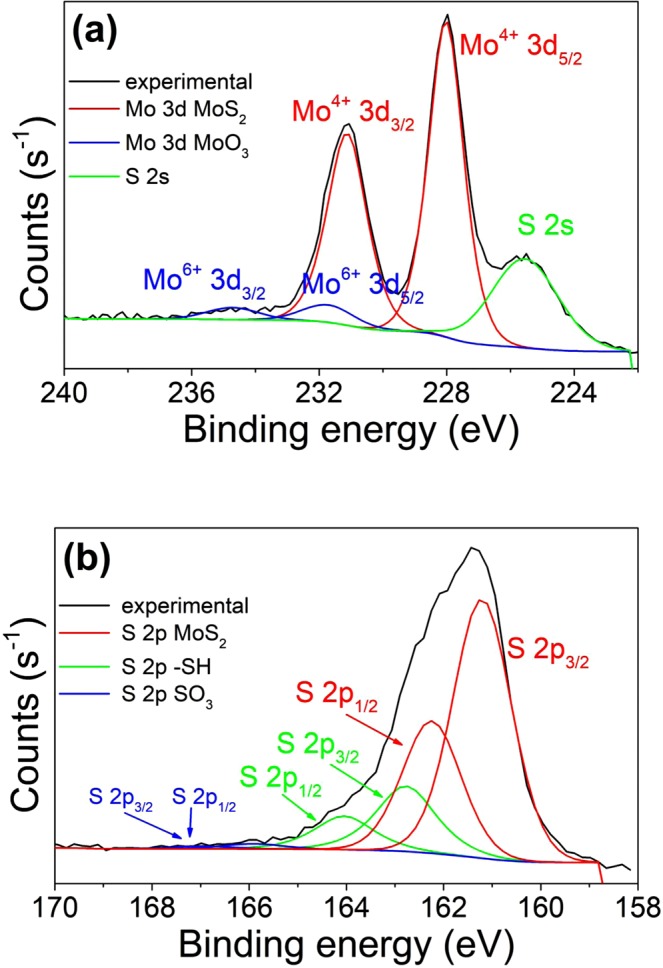
The chemical shifts of (**a**) Mo 3d and (**b**) S 2p core-level binding energies XPS spectra of a MoS_2_ layer grown on microcrystalline CVD diamond film. The overall spectra are deconvoluted into individual components as depicted in the spectrum.

Chemical states and compositions of the as-prepared MoS_2_ layers were analyzed by X-ray photoelectron spectroscopy (XPS). A typical spectrum taken from a MoS_2_ layer grown from a 3 nm Mo film is shown in Fig. 5. The Mo 3d spectral region exhibits two characteristic emission peaks at 231.1 eV (Mo 3d^3/2^) and 228 eV (Mo 3d^5/2^). The measured Mo 3d binding energies match the chemical shifts of the Mo^4+^ state corresponding to MoS_2_^60^. Additionally, the S 2 s peak at the binding energy of 225.7 eV corresponding to MoS_2_ is also observed^60^ in this spectral region. The Mo 3d spin–orbit energy splitting of 3.1 eV is in a good agreement with the previous observations^61,62^. The Mo 3d core-level shift corresponding to the elemental Mo^0^ was not observed. However, a limited contribution of MoO_3_ phase was identified at the binding energies of 231.8 eV (Mo 3d^5/2^) and 234.8 eV (Mo 3d^3/2^) as shown in Fig. 5a. The observed chemical shifts of S 2p core-level states (S 2p^3/2^ and S 2p^1/2^) correspond to MoS_2_ (161.3 and 162.3 eV) and SO_3_ (166.2 and 167.6 eV).

A contribution of the thiol group (-SH) binding states is observed at 162.7 and 164.2 eV. Likely, thiol groups have partially substituted hydrogen atoms terminating the dangling bonds on the diamond surface.

Quantification of peak areas gives the atomic concentration of S and Mo in the sample. The percentage of Mo and S atoms bound in the states corresponding to MoS_2_ is 93% and 77%, respectively. About 21% of sulfur atoms bind to hydrogen in the thiol group.

The calculated S/Mo ratio varies from 2.2 to 2.5 and correlates with the thickness of the deposited Mo films being larger for thinner films. It is important to note that no signal due to molybdenum carbide, Mo_2_C, was detected in XPS spectra^58,59^ which is in agreement with Raman measurements.

**Figure 6:**
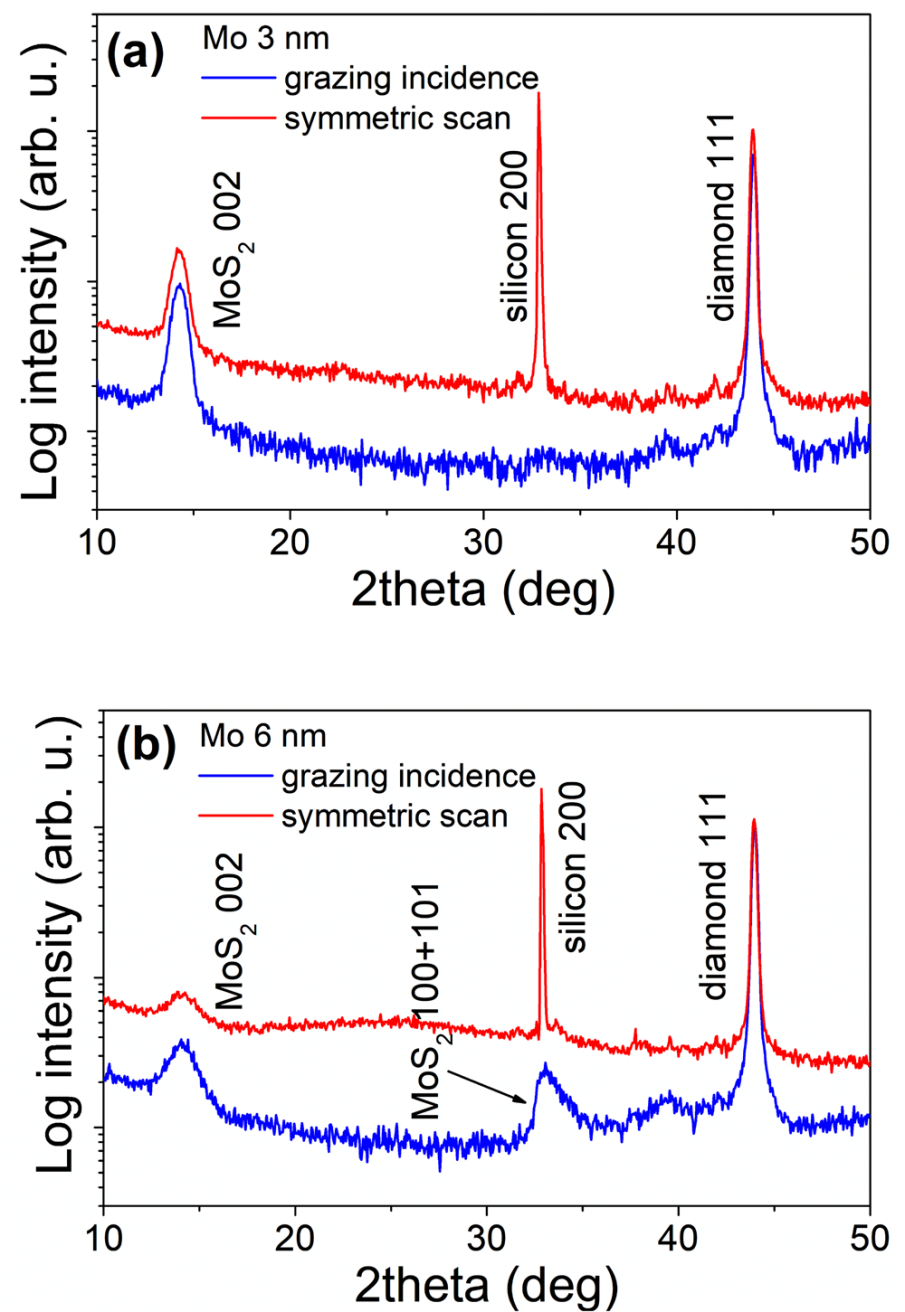
XRD patterns in symmetric (red line) and grazing-incidence (blue line) geometry of MoS_2_ layers prepared from (**a**) 3 nm and (**b**) 6 nm thick Mo films. Diffraction peaks of MoS_2_, silicon and diamond are indexed in the figures.

The crystalline structure of the MoS_2_ layers grown on the microcrystalline CVD diamond substrates was studied by XRD. We did not register any MoS_2_ diffraction peaks in the layers grown from 1 nm thick Mo films. We suppose that the total amount of the MoS_2_ is below the detection limit of our diffractometer setup. The symmetric and grazing incidence XRD patterns of the MoS_2_ layers prepared from 3 nm and 6 nm thick Mo film are shown in Fig. 6a,b, respectively. XRD confirms the presence of MoS_2_ hexagonal 2H phase as similarly identified by Raman.

The comparable intensities of 111 diamond diffractions measured in symmetric and grazing-incidence geometries confirm untextured polycrystalline diamond film. On the other hand, the significant intensity changes of 002 MoS_2_ diffractions found between the two employed XRD measurement geometries imply the growth of a textured MoS_2_ film on the top of polycrystalline diamond substrate. As shown in Fig. 6, the intensity of the 002 diffraction peak is weaker for MoS_2_ layers grown from a thicker Mo film than that for the 3 nm Mo one. This means that increasing the Mo thickness the crystallographic orientation of the MoS_2_ layers with respect to diamond substrate is changing.

Conversely, the change of the MoS_2_ layer alignment makes other diffractions detectable as confirmed by an appearance of the 100 and 101 diffractions in a grazing incidence measurement. In order to explain these observations, we additionally performed grazing-incidence wide-angle X-ray scattering (GIWAXS) measurements. This method is suitable for studying the crystallographic orientation of thin polycrystalline films^63^. In contrast to HRTEM (high resolution transmission electron microscopy), which is a rather local probe, GIWAXS technique provides a statistical average over the whole sample area. Moreover, no special sample preparation is necessary.

**Figure 7:**
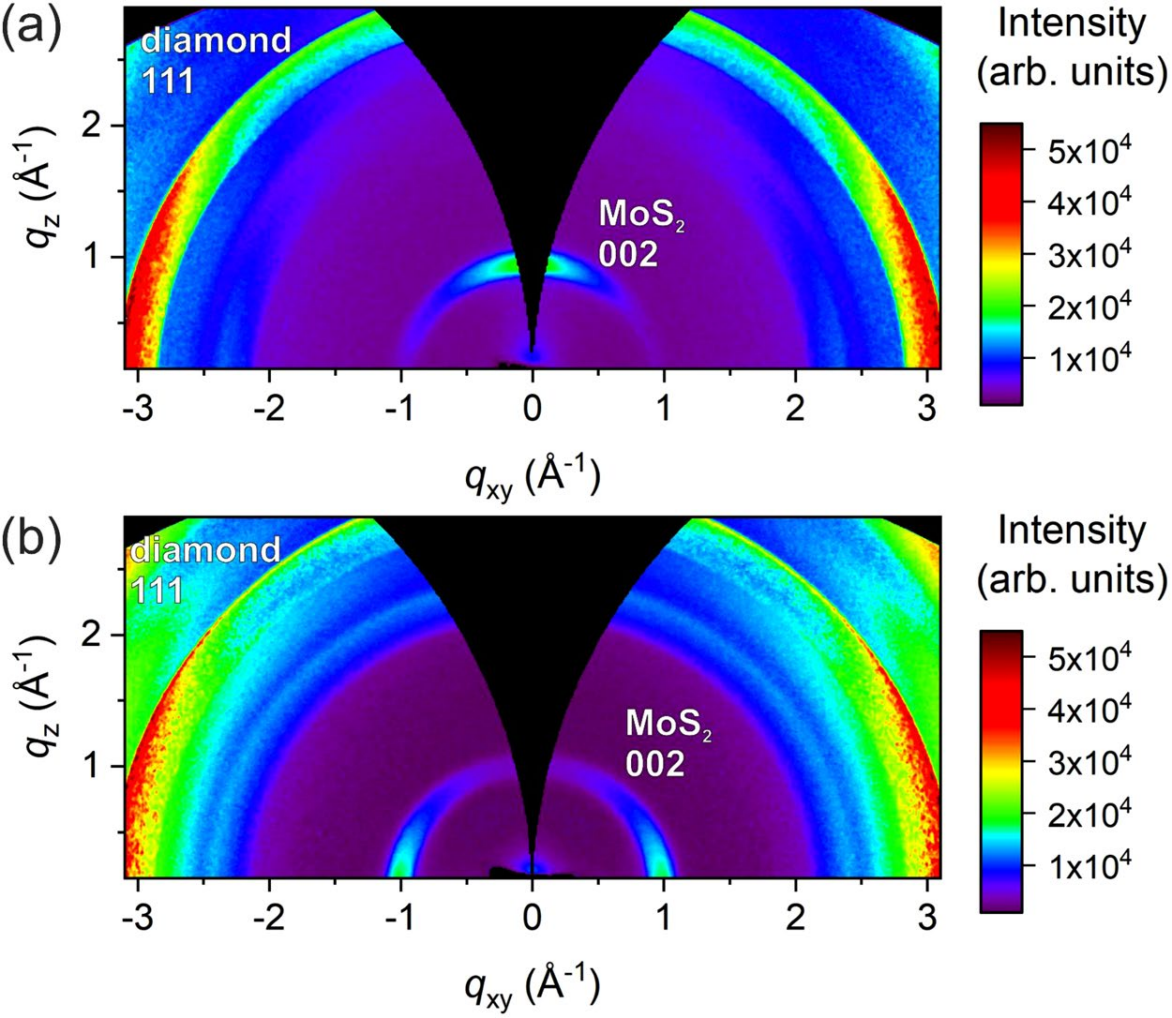
GIWAXS reciprocal space maps of MoS_2_ layers prepared from Mo films with the nominal thickness of (**a**) 3 nm and (**b**) 6 nm The intensities are normalized to a diamond 111 diffraction.

The GIWAXS reciprocal space maps of the sulfurized 3 and 6 nm Mo films are shown in Fig. 7a,b, respectively. The diffraction ring at *q* ~ 3 Å^−1^ belongs to 111 diffraction of diamond phase. The most intense 002 diffraction of MoS_2_ is located at 1 Å^−1^. The two less intense 100 and 103 diffractions can be found at 2.3 and 2.7 Å^−1^, respectively. The 101 diffraction merges with 100 on the scale used in the figure.

The MoS_2_ layers exhibit a uniaxial texture with the crystallographic c-axis aligned along the substrate surface normal (Fig. 7a) for layers grown from 3 nm thick Mo. On the other hand, the c-axis is perpendicular to the surface normal (Fig. 7b) for layers grown from 6 nm Mo as validated by the oriented 002 partial diffraction rings. The apparent moderate orientation degree of crystallographic c-axis has an origin in large angular spread of underlying polycrystalline diamond facets as explained below.

The influence of initial Mo thickness on the crystallographic orientation was already observed in the case of MoS_2_ films prepared on flat substrates^45,47,49,50^. The results of our GIWAXS measurements validate the same behavior also for MoS_2_ layers grown on rough CVD diamond.

**Figure 8:**
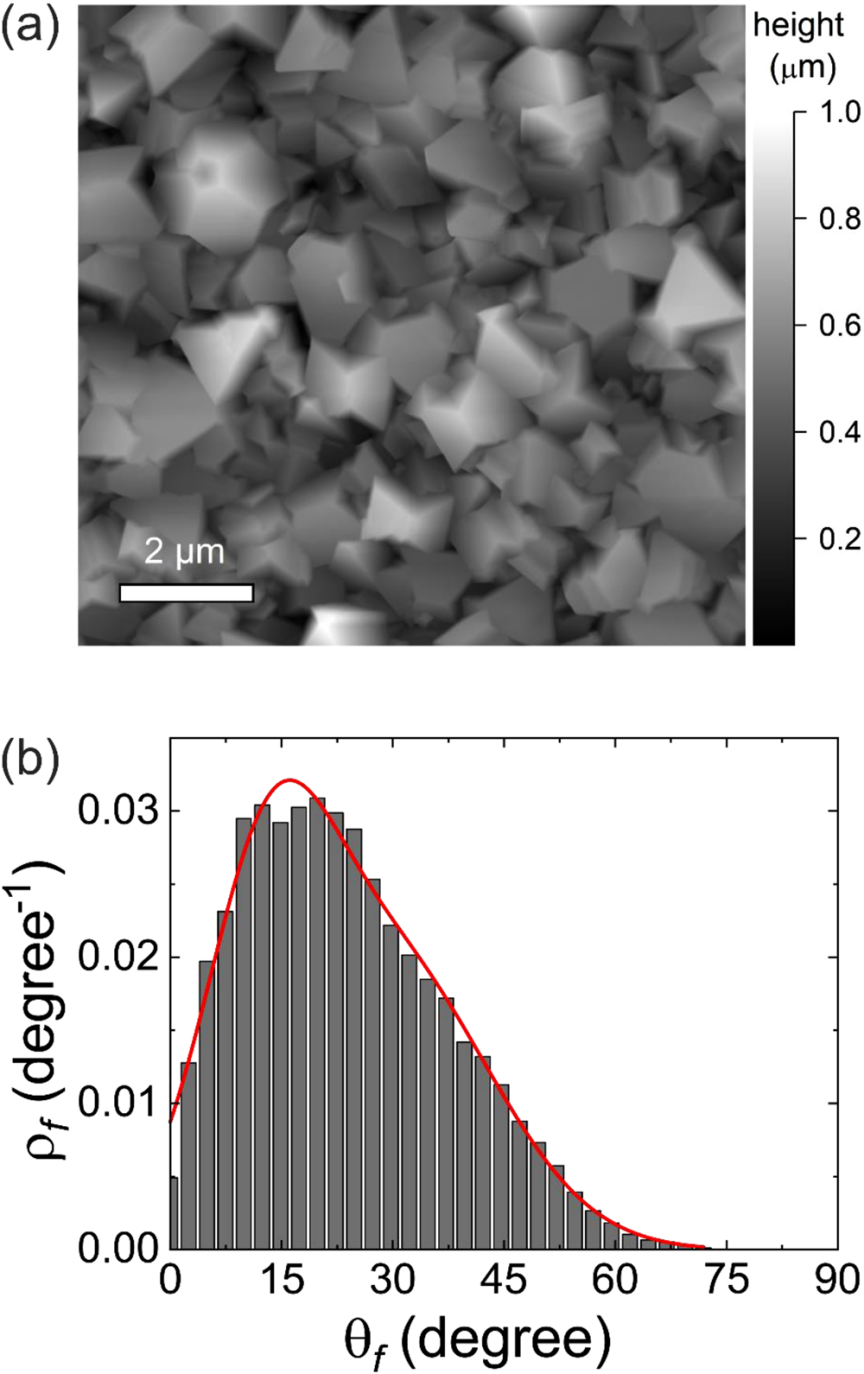
(**a**) AFM micrograph of the microcrystalline CVD diamond surface and (**b**) the corresponding distribution of inclinations of the diamond facets. The red curve shows the bi-Gaussian fit of the angular distribution of diamond facets.

For deeper understanding, we have also used AFM to determine the angular distribution of the diamond facets supporting the thin MoS_2_ layers. Figure 8a shows the AFM image of the microcrystalline CVD diamond film. Figure 8b shows the calculated distribution of the inclination angles of diamond facets^64^. The θ_f_ angle, plotted on the x-axis, corresponds to the angle between the normal of the substrate and the normal of diamond facets, i.e. the angle between the substrate plane and the surface of the diamond. The y-axis represents the normalized angular density of diamond facets having the inclination angle θ_f_. The angular distribution is well described by the bi-Gaussian distribution function (Fig. 8b) having the maximum at approximately 15°.

It is obvious, that although the diamond films have no preferential crystallographic orientation, the measured angular deviations of the diamond facets from the substrate plane will have a pronounced effect on the GIWAXS texture measurements. Indeed, this is in a good agreement with the 002 diffracted intensity distributions in the GIWAXS measurements for the vertically and horizontally grown MoS_2_ layers (Fig. 7). The angular distribution of the diamond facets gives rise to smeared intensity distributions along the 002 diffraction ring with the maximum at q_z_ and q_xy_ for the horizontally (3 nm Mo) and vertically (6 nm Mo) grown MoS_2_ layers, respectively.

**Figure 9:**
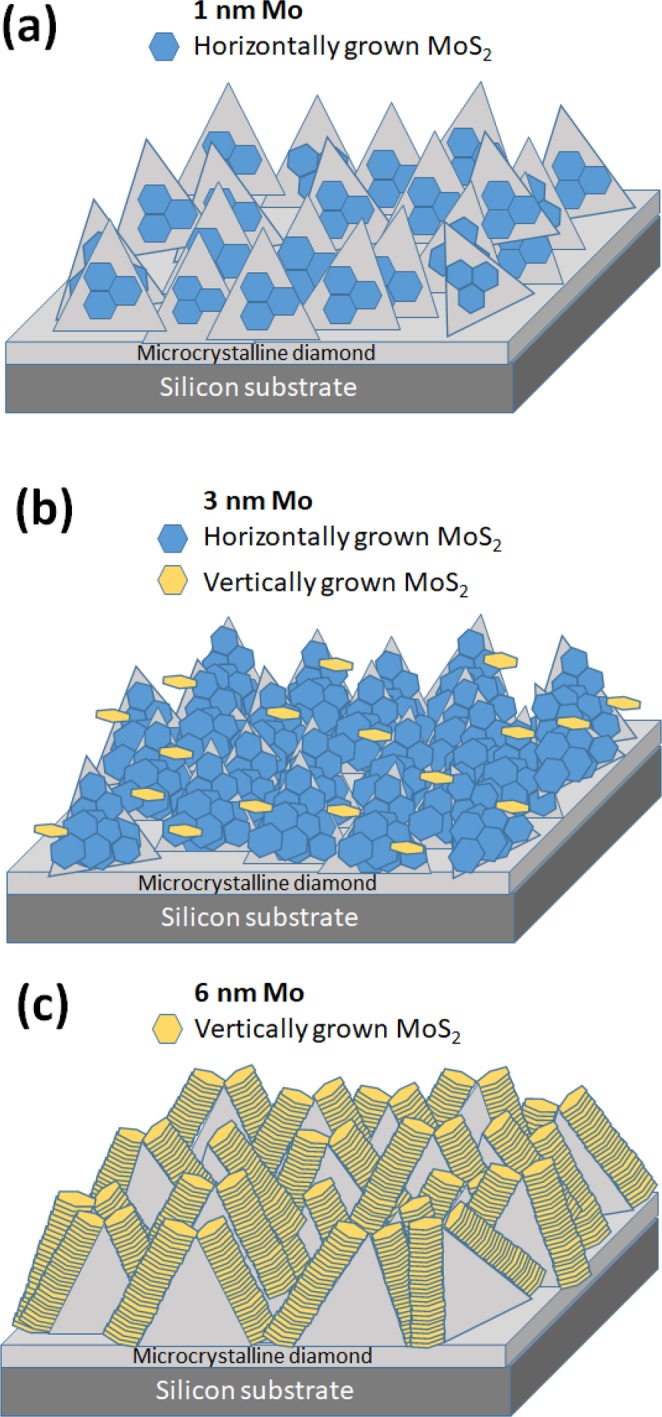
Schematic representation of MoS_2_ layers grown from films of different Mo thickness on the top of the microcrystalline CVD diamond films.

Based on SEM and GIWAXS results, we propose the following growth modes for the formation of a MoS_2_ film. Figure 9 shows a schematic representation of anticipated growth of MoS_2_ layers depending on the initial thicknesses of the Mo seeding. Using the 1 nm Mo, we observed a dominant horizontal growth of discontinuous MoS_2_ layers as concluded from SEM and Raman measurements (Fig. 9a). As the Mo film thickness increases to 3 nm, the diamond substrate is horizontally grown with MoS_2_ layers covering all its surface. To note, we identified also certain areas where a combination of the simultaneous horizontal and vertical growth was evidenced (Fig. 9b). This is in a good agreement with the results obtained from GIWAXS measurements. Finally, employing the Mo thickness of 6 nm induced the dominance of vertically grown MoS_2_ layers (Fig. 9c).

Up to now, a detailed growth mechanism of the MoS_2_ films prepared by the sulfurization of pre-deposited Mo films is still under discussion. Firstly, the sulfur starts to form the MoS_2_ layers on the top of the Mo films. In such a case, the conventional bottom-up mechanism (as in the case of one-step methods e.g. pulsed laser deposition^51^) is not applicable. When the Mo thickness exceeds a critical threshold thickness, 6 nm in our case, the vertical growth of MoS_2_ occurs exclusively. Jung *et al.*^45^ have found that the thickness of the initial Mo film is indeed the critical parameter that dictates the preferential orientation of the MoS_2_ layers. However, the identification of exact threshold thickness is still uncertain. Even though the nominal thickness of the as-deposited Mo films was 1, 3 and 6 nm, the surface morphology of the diamond film (Fig. 2a) may locally decrease the thickness of the Mo films^65^. The self-shadowing effect is naturally present due to the uneven substrate features and as a result of a directional Mo vapor flux^66^. Consequently, the thickness of a deposited Mo film varies locally due to preferential deposition of the incident Mo atoms on higher surface points^67^. To distinguish the value more certainly, atomically flat substrates has to be used.

In sum our study confirms that the MoS_2_ growth on the well-faceted and rough diamond thin films can be simply tailored just by tuning the thickness of a pre-deposited Mo films. This technological issue can be crucial especially for applications where vertically aligned MoS_2_ layers are required^1^.

## Conclusion

Few-layer MoS_2_ were prepared on microcrystalline CVD diamond substrates by sulfurization of pre-deposited Mo films. In the course of sulfurization, Mo coated substrates and sulfur powder were placed close to each other in a one-zone furnace and exposed to the same temperature so an additional control over the sulfur temperature was not needed. As a results of this particular experimental design, the sulfur-rich environment during the process and diffusion of sulfur into molybdenum at temperatures below that required for formation of molybdenum carbide prevents the formation of the latter at the Mo-diamond interface. This finding may open a way for growing MoS_2_ layers on substrates which are otherwise susceptible to a chemical reaction with molybdenum. We have also demonstrated a horizontal and vertical growth of MoS_2_ layers, in terms of crystallographic *c*-axis orientation, by tuning the Mo film thickness. The combination of diamond unique properties and MoS_2_ ultra-thin layers with a tunable crystallographic orientation can offer material properties relevant for a wide range of applications.
